# Birth outcomes associated with maternal antiglaucoma medication exposure: a systematic review and meta-analysis

**DOI:** 10.3389/fmed.2026.1872415

**Published:** 2026-07-08

**Authors:** Hanying Fan, Yiji Zhang, Lin Jing, Yu He, Suzhen Wang, Liuzhi Zeng

**Affiliations:** 1 Department of Ophthalmology, Chengdu Integrated TCM & Western Medicine Hospital, Chengdu, Sichuan, China; 2 Department of Obstetrics and Gynecology, Chengdu Integrated TCM & Western Medicine Hospital, Chengdu, Sichuan, China

**Keywords:** adverse birth outcomes, antiglaucoma medication, congenital anomalies, glaucoma during pregnancy, low birth weight, preterm birth

## Abstract

**Background:**

Antiglaucoma medications are indispensable for preserving maternal vision, yet the associations between their use and adverse birth outcomes remain inconclusive.

**Method:**

The meta-analysis was conducted in accordance with the PRISMA 2020 and MOOSE guidelines. We searched Web of Science, PubMed, Embase, and ScienceDirect for eligible comparative studies. The outcomes were low birth weight (LBW), preterm birth (PB) and congenital anomalies (CA). The pooled results are expressed as odds ratios (ORs) with 95% confidence intervals (95% CIs). Influential publication was determined by performing sensitivity analysis. In addition, the potential sources of heterogeneity were examined by using subgroup analyses. Publication bias was assessed using funnel plots and Begg’s and Egger’s tests.

**Results:**

The 8 included studies (11 datasets) were published between 2004 and 2024 and included 3,713 pregnant women with glaucoma. All the included studies were of good to excellent quality. Maternal use of antiglaucoma medications was associated with an increased risk of LBW (OR = 2.12, 95% CI = 1.46 to 3.08, *p* < 0.001). However, no significant associations were found for the risks of PB (OR = 1.58, 95% CI = 0.81 to 3.07, *p* = 0.180) or CA (OR = 1.07, 95% CI = 0.24 to 4.77, *p* = 0.933). Subgroup analysis revealed that mean maternal age was potential source of heterogeneity for the risk of CA (interaction *p*-value <0.001). No influential publications or significant publication bias was detected across the studies.

**Conclusion:**

Maternal use of antiglaucoma medications was potentially associated with an increased risk of LBW. In contrast, the evidence regarding PB and CA risk remains inconclusive. However, the preservation of maternal visual function remains a critical priority in certain cases.

**Systematic review registration:**

https://www.crd.york.ac.uk/PROSPERO/recorddashboard, Identifier CRD420261365100.

## 1 Introduction

Glaucoma represents the foremost etiology of irreversible visual impairment globally, with primary open-angle glaucoma (POAG) constituting the predominant subtype. Epidemiological data indicate that the prevalence of POAG is 0.48, 0.42, and 0.73% in women aged 15–24, 25–34, and 35–44 years, respectively (1). However, glaucoma during gestation is uncommon and no definitive guidelines have been established to date. The scarcity of large-scale clinical data on drug safety in this population has hindered the development of standardized protocols (2). Therefore, the management of glaucoma in pregnant patients presents a significant clinical dilemma, where the fetal safety profile of therapeutic interventions is of paramount importance.

Although intraocular pressure (IOP) typically decreases during pregnancy, some patients experience disease progression and require medical or surgical intervention (3). Furthermore, pregnancy is associated with various reversible visual field alterations, which may manifest as bitemporal or concentric constriction and an expansion of the physiologic blind spot (4). Consequently, despite the paucity of robust clinical data regarding the use of glaucoma medications in pregnant patients, pharmacological intervention often remains imperative to ensure adequate disease control during gestation (5). However, this therapeutic necessity contrasts with current clinical confidence. Survey data revealed that although 26% of clinicians had previously managed glaucoma among pregnant women, the appropriate therapeutic approach for this specific population remains uncertain (2).

The management of glaucoma during pregnancy presents a significant therapeutic dilemma, primarily because of the potential teratogenic risks and adverse systemic effects of antiglaucoma medications on fetal development. Brimonidine is the only IOP-lowering medication classified as Category B, which implies that animal studies have not found a risk to the fetus, although there are no adequate and well-controlled studies in pregnant women (6). It has previously been recommended as a preferred therapeutic option for glaucoma during the first and second trimesters of pregnancy, with the suggestion to discontinue its use in the third trimester because of theoretical safety concerns (7). In contrast, all other IOP-lowering agents, including prostaglandin analogs (PGs), beta-blockers (BBs), and carbonic anhydrase inhibitors, are classified as Category C (6). Hypothetically, PGs can traverse the blood–placenta barrier and precipitate uterine contractions, thereby inducing preterm labor or abortion (8). Furthermore, exposure to PGs during the first trimester is hypothesized to increase the risk of neonatal anomalies, with the underlying mechanism attributed to the induction of fetal cerebral ischemia (9). Given these concerns, empirical evidence regarding the safety of glaucoma medications has been largely extrapolated from animal models or small case series.

However, the acquisition of further human data remains imperative to better elucidate the fetal safety profile of these agents. To address this gap in evidence, we conducted a meta-analysis to investigate the associations between maternal use of antiglaucoma medications and adverse birth outcomes.

## 2 Methods

The study was in line with the Preferred Reporting Items for Systematic Reviews and Meta-Analysis (PRISMA) and MOOSE guidelines (10, 11). The protocol for this systematic review and meta-analysis was registered on PROSPERO (ID: CRD420261365100).

### 2.1 Eligibility criteria

This meta-analysis includes studies on the birth outcomes associated with antiglaucoma medications during pregnancy. The outcomes were low birth weight (LBW), preterm birth (PB) and congenital anomalies (CA). To ensure consistency across the included studies, the outcomes were strictly defined according to standard clinical criteria. Preterm birth was defined as delivery before 37 completed weeks of gestation, and low birth weight was defined as a birth weight of less than 2,500 g. When studies used slightly different definitions or thresholds, we extracted the data that most closely matched our predefined criteria. Although some included studies were identified as case series, they were eligible for this comparative meta-analysis because they reported complete follow-up records with distinct information on maternal medication exposure. Studies that met one of the following criteria were excluded: (1) laboratory-based research, (2) non-English language, or (3) full text unavailable.

### 2.2 Search strategy

The literature search was conducted in accordance with the PICO framework, focusing on pregnant women with glaucoma (P), antiglaucoma medication therapy (I), and birth outcomes (O). The Web of Science, PubMed, ScienceDirect and Embase databases were screened up to April, 2026. In addition, a manual search of reference lists from relevant reviews and meta-analyses was performed to identify eligible studies. The search strategy utilized the following query: (Glaucoma or Glaucomas) and (Pregnancy or Pregnant or Gestation or Gestational or Pregnancies). The exact search strings and syntax for each database are detailed in Supplementary Table S1.

### 2.3 Study selection

After the studies were retrieved from the databases, the records were deduplicated using EndNote software. Two reviewers independently screened the titles and abstracts for relevance to the study objectives. Finally, the full texts of potentially eligible studies were assessed against the inclusion and exclusion criteria. Any discrepancies between the reviewers were resolved via discussion or by consulting a third reviewer.

### 2.4 Quality assessment

Two reviewers independently assessed the risk of bias for the observational studies with the Newcastle–Ottawa Scale (NOS) checklist (12). The NOS checklist has 7 dedicated items, each of which receives 1 point, except for the comparison item, which can receive a maximum of 2 points. In addition, we used the Joanna Briggs Institute (JBI) critical appraisal checklist to assess the methodological quality of the case series (13). Specifically, we focused on items related to the validity of exposure measurement (glaucoma medication use) and the completeness of outcome reporting (birth outcomes), which were critical for establishing internal comparisons. Any discrepancies were resolved by consulting a third reviewer.

### 2.5 Data extraction and synthesis

Data extraction was performed independently by two reviewers, with discrepancies resolved through discussion or adjudication by a third reviewer. A standardized data collection form was used to extract the following variables: first author, publication year, country, study duration, study design, sample size, mean maternal age, and antiglaucoma agent. Within each case series, we extracted data to stratify participants into exposed and unexposed groups on the basis of whether glaucoma medications were used during pregnancy. In this situation, these data allowed for an internal comparison between groups rather than serving as single-arm descriptions.

The pooled results are expressed as odds ratios (ORs) with 95% confidence intervals (95% CIs). Statistical heterogeneity was evaluated using the I^2^ statistic and Cochran’s Q test, where values of 25, 50, and 75% were interpreted as low, moderate, and high heterogeneity, respectively. A random-effects model was employed when moderate -to- high heterogeneity was detected; otherwise, a fixed-effects model was applied. Influential publication was determined by performing sensitivity analysis. In addition, potential sources of heterogeneity were identified by subgroup analysis. Publication bias was assessed by using a funnel plot and Begg’s and Egger’s tests.

### 2.6 Statistical analysis

Statistical analyses were performed using Stata 16 software. The “metan,” “metaninf,” “metafunnel,” and “metabias” commands were utilized to conduct the pooled analyses, sensitivity analyses, and publication bias assessments, respectively. All statistical tests were two-tailed, with a *p* value < 0.05 considered to indicate statistical significance.

## 3 Results

This study presents a comprehensive meta-analysis exploring the risk of adverse birth outcomes associated with antiglaucoma medication exposure during pregnancy, and investigating potential factors associated with birth outcomes.

### 3.1 Study selection

**Figure 1:**
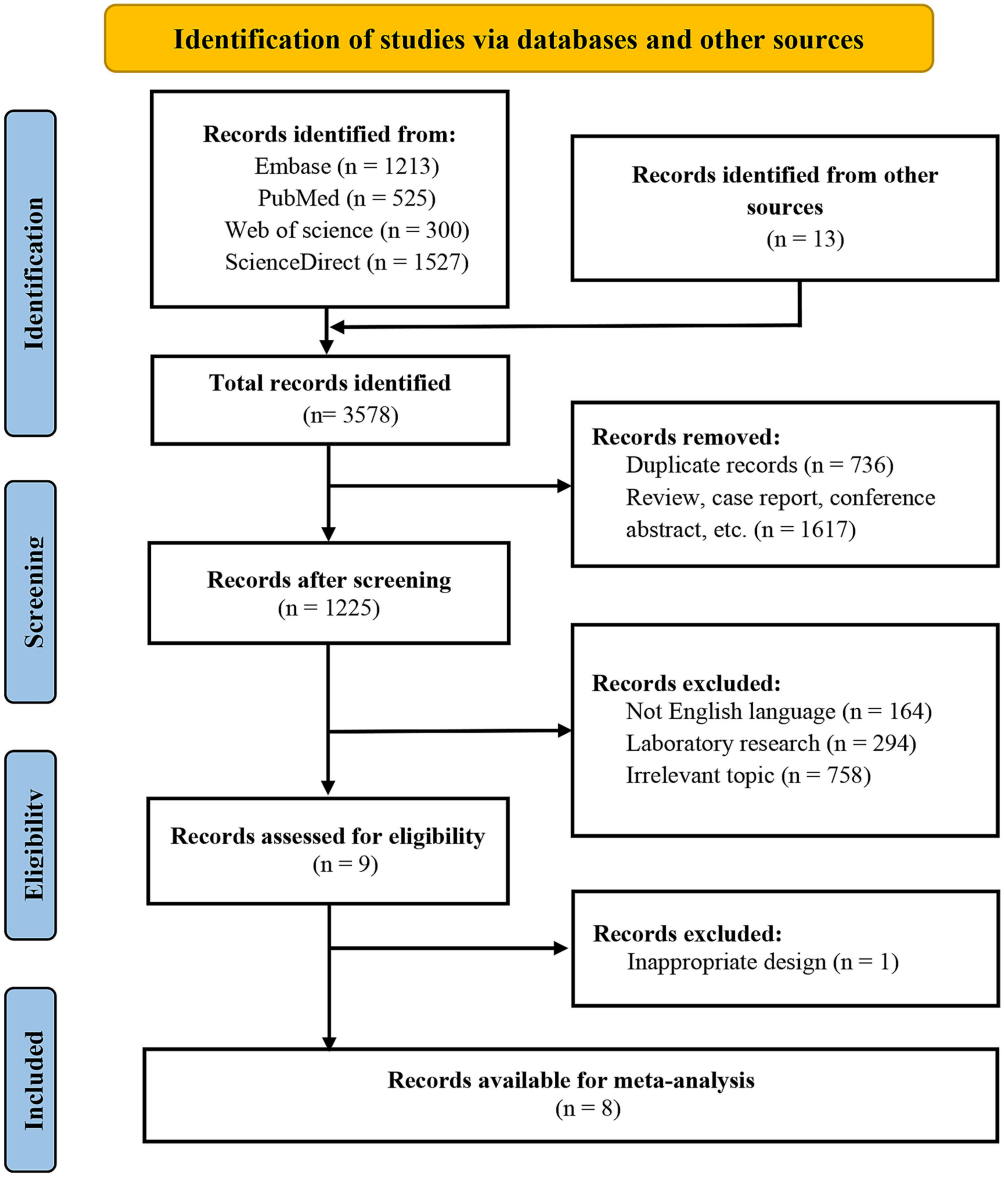
PRISMA flowchart of the 8 included studies.

The study selection process is presented in Figure 1. An initial search yielded 3,565 records from electronic databases, supplemented by 13 additional studies identified through manual screening of reference lists. After 736 duplicates and 1,617 irrelevant records (e.g., reviews and case reports) were removed, 1,225 records were screened. After the exclusion of 164 non-English studies, 294 laboratory studies, and 758 studies with irrelevant topics, 9 full-text articles were assessed for eligibility. Ultimately, 8 studies were included in the final analysis (14–21).

### 3.2 Study characteristics and quality assessment

The 8 studies included (5 cohort studies and 3 case series) were published between 2004 and 2024. These studies enrolled a total of 3,713 patients, with sample sizes ranging from 8 to 2,196 participants. The mean age of the pregnant women ranged from 28 to 35 years. A comprehensive summary of the study characteristics is provided in Table 1.

**Table 1:** Summary characteristics of the 8 included studies.

Author	Year	Country	Study duration	Study design	Simple size	Mean age (year)	Antiglaucoma agent	Outcome
Brauner, S., et al. (14)	2006	USA	2003–2006	Case series	15	30	Other	LBW, PB, CA
De Santis, M., et al. (15)	2004	Italy	2000–2002	Case series	9	31	PGA	LBW, PB, CA
Hashimoto, Y., et al. (16)	2021	Tokyo	2005–2018	Cohort	798	35	BB, PGA	LBW, PB, CA
Ho, J., et al. (17)	2009	China	1996–2003	Cohort	2,196	30	BB, Other	LBW
Kaufman, A., et al. (18)	2024	USA	2021–2022	Cohort	295	29	BB, PGA, Other	LBW, PB, CA
Mendez, C., et al. (19)	2012	Spain	2002–2010	Case series	8	31	PGA	LBW, PB
Pellegrino, M., et al. (20)	2018	Italy	2003–2015	Cohort	262	30	BB, Other	PB
Razeghinejad, M., et al. (21)	2010	Iran	2002–2006	Cohort	30	28	Other	LBW

BB, β-blocker; CA, congenital anomalies; LBW, low birth weight; NOS, Newcastle-Ottawa Scale; PB, preterm birth; PGA, prostaglandin analogue.

Methodological quality was assessed using the NOS checklist for the 5 cohort studies (16–18, 20, 21). On the basis of the quality score, these cohort studies scored 7 or 8 points, and no study was excluded (Supplementary Table S2). The 3 case series (14, 15, 19) included in this study were assessed using the Joanna Briggs Institute (JBI) Critical Appraisal Checklist, and the results are presented in Supplementary Figure S1.

### 3.3 Adverse birth outcomes associated with antiglaucoma medication exposure during pregnancy

#### 3.3.1 Maternal antiglaucoma medication exposure was associated with an increased risk of LBW

**Figure 2:**
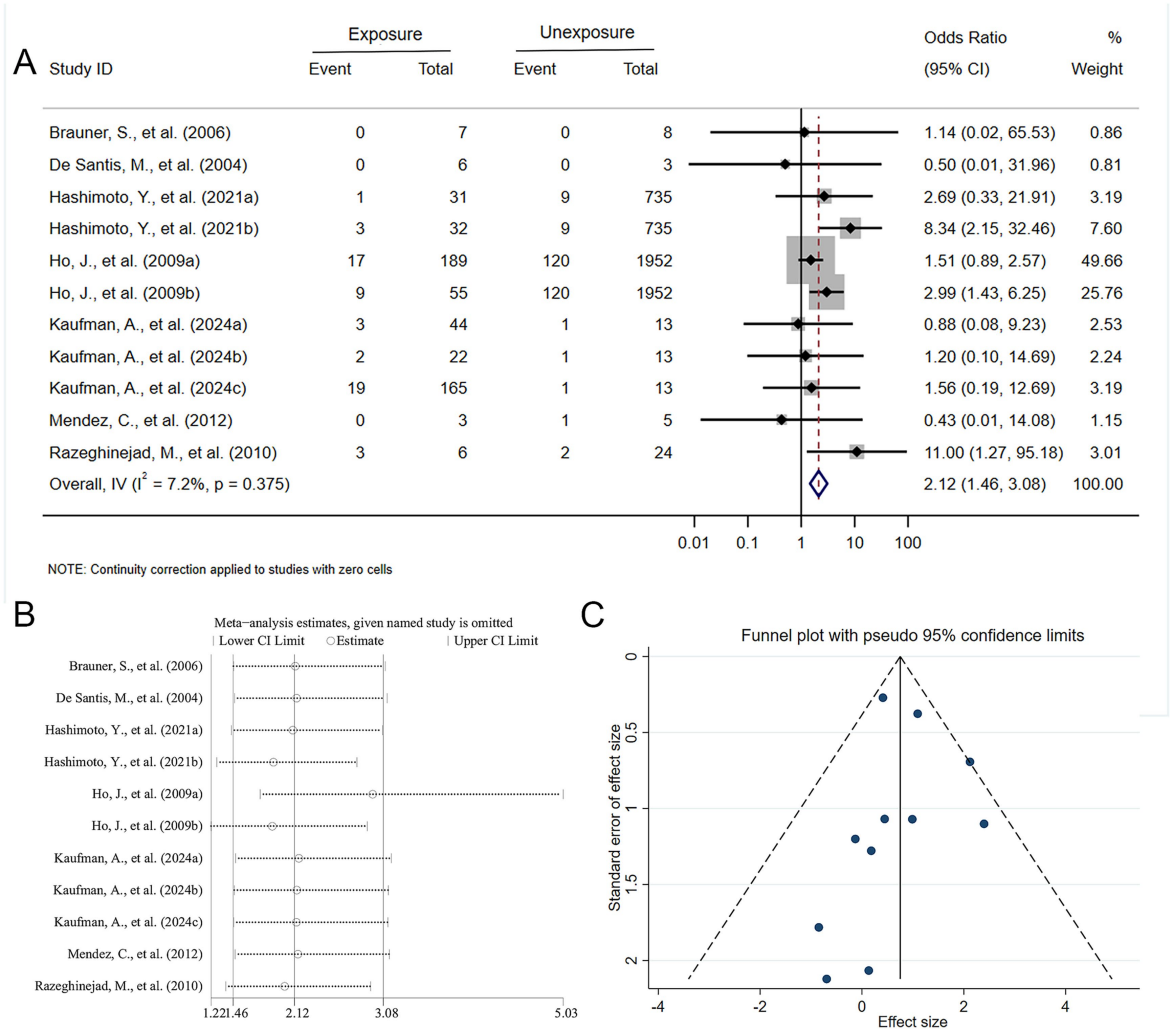
Maternal antiglaucoma medication exposure was associated with an increased risk of LBW. **(A)** Forest plot of the risk of LBW associated with maternal antiglaucoma medication exposure; **(B)** Sensitivity analysis for the risk of LBW; **(C)** Funnel plot for assessing publication bias.

A fixed-effects meta-analysis of 7 studies (14–19, 21) revealed that maternal use of antiglaucoma medications was significantly associated with an increased risk of LBW (OR = 2.12, 95% CI = 1.46 to 3.08, *p* < 0.001). The analysis revealed low heterogeneity (I^2^ = 7.2%, *p* = 0.375) (Figure 2A). Influence analysis indicated that no single study disproportionately affected the pooled estimate (Figure 2B). Furthermore, no significant publication bias (Figure 2C; Begg’s test: z = −1.09, *p* = 1.000; Egger’s test: *t* = 0.00, *p* = 0.999) was detected.

#### 3.3.2 No significant link between PB risk and maternal glaucoma medication use

**Figure 3:**
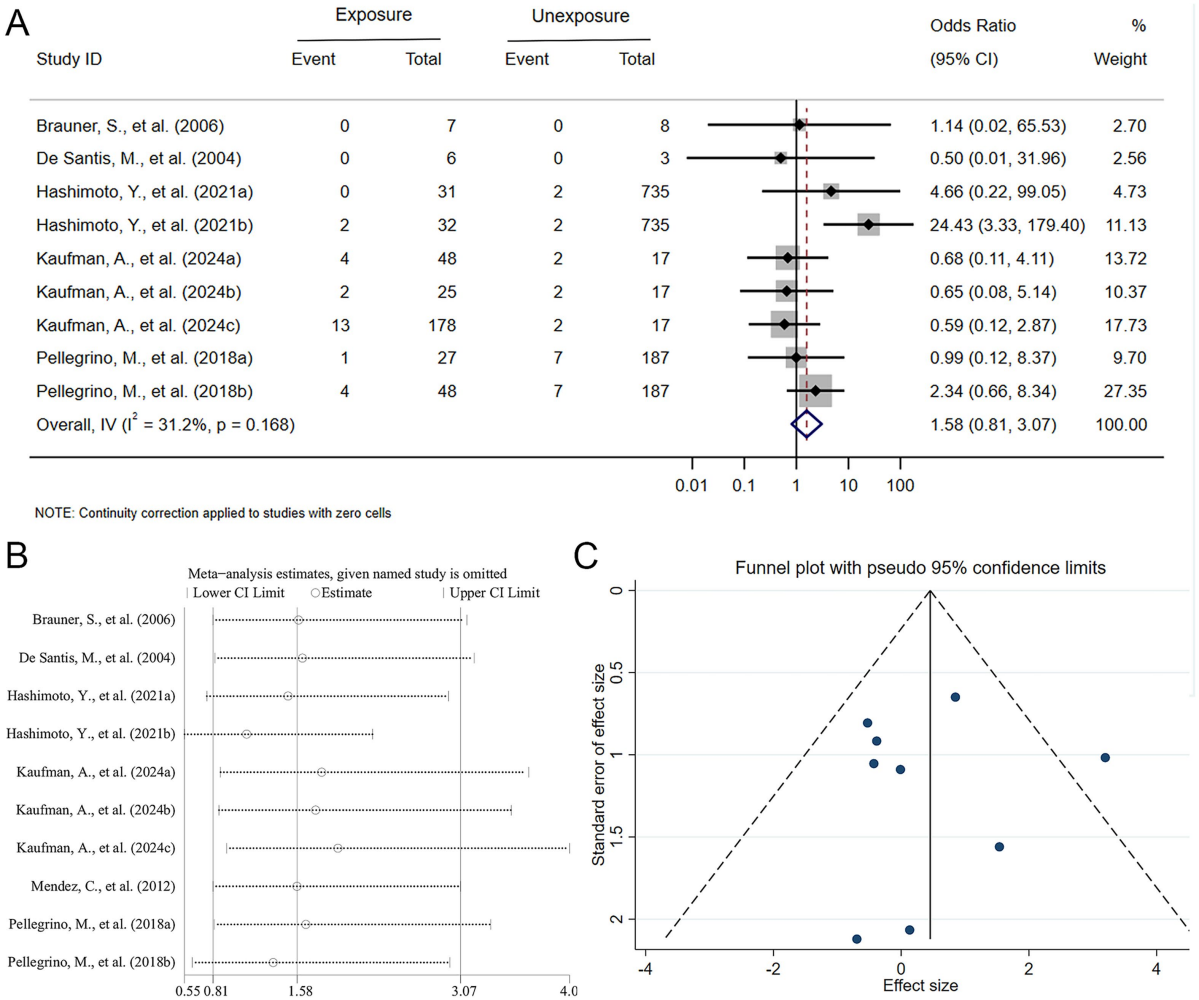
There was no significant link between PB risk and maternal glaucoma medication use. **(A)** Forest plot of the risk of PB associated with maternal antiglaucoma medication exposure; **(B)** Sensitivity analysis for the risk of PB; **(C)** Funnel plot for assessing publication bias.

A fixed-effects meta-analysis of 6 studies (14–16, 18–20) revealed no significant association between maternal antiglaucoma medication use and the risk of PB (OR = 1.58, 95% CI = 0.81 to 3.07, *p* = 0.180). The analysis revealed low heterogeneity (I^2^ = 31.2%, *p* = 0.168) (Figure 3A). Influence analysis indicated that no single study disproportionately affected the pooled estimate (Figure 3B). Furthermore, no significant publication bias (Figure 3C; Begg’s test: z = 0.31, *p* = 0.754; Egger’s test: *t* = −0.10, *p* = 0.921) was detected.

#### 3.3.3 No significant association between CA risk and maternal antiglaucoma medication exposure

**Figure 4:**
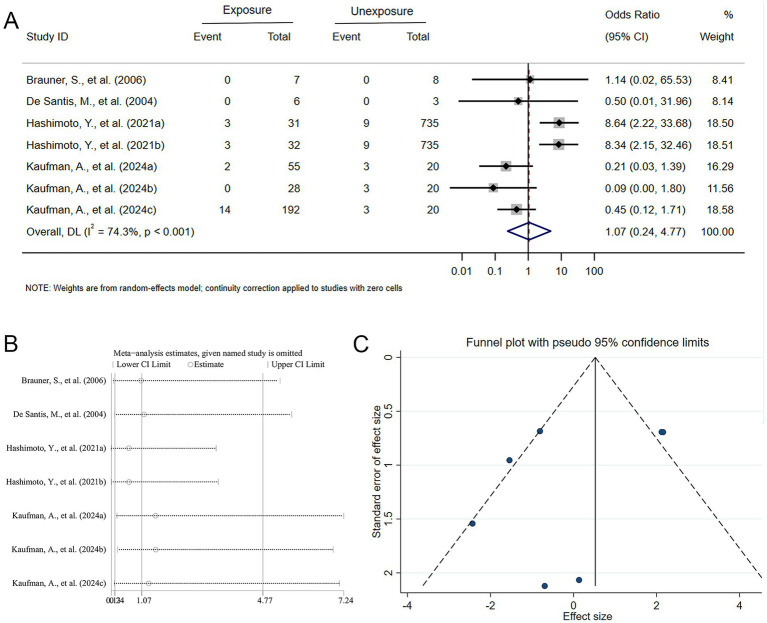
No significant association between CA risk and maternal antiglaucoma medication exposure. **(A)** Forest plot of the risk of CA associated with maternal antiglaucoma medication exposure; **(B)** Sensitivity analysis for the risk of CA; **(C)** Funnel plot for assessing publication bias.

A random-effects meta-analysis of 4 studies (14–16, 18) revealed no significant association between maternal antiglaucoma medication use and the risk of CA (OR = 1.07, 95% CI = 0.24 to 4.77, *p* = 0.933). The analysis revealed high heterogeneity (I^2^ = 74.3%, *p* < 0.001) (Figure 4A). Influence analysis indicated that no single study disproportionately affected the pooled estimate (Figure 4B). The results of the subgroup analysis revealed that the mean maternal age (interaction *p*-value <0.001, Supplementary Figure S2A) was a potential source of heterogeneity, whereas the study design (interaction *p*-value = 0.829, Supplementary Figure S2B) and antiglaucoma agent (interaction *p*-value = 0.821, Supplementary Figure S2C) were not. Furthermore, no significant publication bias (Figure 4C; Begg’s test: z = 0.00, *p* = 1.000; Egger’s test: *t* = −1.02, *p* = 0.306) was detected. However, given the relatively small number of included studies, the statistical power to detect potential bias is limited, and these results should be interpreted with caution.

## 4 Discussion

Glaucoma, recognized as the leading cause of irreversible blindness worldwide, presents a unique management paradigm in the obstetric population (1). Although the diagnosis of glaucoma during pregnancy is infrequent, patients with preexisting glaucoma may occasionally conceive. The IOP in pregnant women with ocular hypertension often tends to decrease, and a subset of patients continue to exhibit uncontrolled IOP, necessitating therapeutic intervention to prevent optic nerve impairment during gestation (22). This physiological complexity is further compounded by anatomical changes. For instance, Weinreb et al. employed ultrasound pachymetry to evaluate central corneal thickness in a cohort of 89 pregnant women. Their analysis revealed a 3% increase in thickness relative to control eyes. Notably, this physiological change was found to be independent of the gestational trimester and was observed to resolve postpartum, returning to baseline levels (23). Critically, a subset of patients exhibit disease progression or fail to achieve target IOP, thereby requiring medical intervention to prevent permanent visual field defects.

Despite these physiological nuances, the clinical management of this condition remains challenging. In a study of pregnant glaucoma patients, most adhered to their established medication protocols, while 34% opted for observation. However, 31% of the respondents were unsure how to manage the contingency of active treatment (2). This clinical equipoise often leads to suboptimal decision-making, placing both maternal vision and fetal safety at risk. This uncertainty highlights a broader issue: many clinicians may not be fully acquainted with the nuances of managing glaucoma in pregnant patients, a knowledge gap attributable to both the low prevalence of the disease among women of childbearing age and the dearth of robust human safety data regarding the therapeutic armamentarium (2). Ultimately, owing to the lack of management guidelines for pregnant women, both clinicians and patients are concerned about the potential adverse effects of therapeutic interventions.

The safety profiles of antiglaucoma medications are primarily categorized on the basis of animal models or small case series, as large-scale human data are lacking. Brimonidine is the only IOP-lowering medication classified as Category B, implying that there is no observed risk in animal studies but inadequate data in pregnant women. Given these teratogenic risks and adverse systemic effects, the medical management of glaucoma in pregnant patients represents a significant clinical dilemma in which fetal safety is of paramount importance. Among the other available therapeutic options, BBs have the most extensive clinical experience. Owing to their established role in the systemic management of hypertension during pregnancy, ophthalmologists and obstetricians exhibit greater confidence in their use. Topical BBs, particularly timolol, represent the predominant therapeutic choice for the management of glaucoma in the obstetric population (24). Theoretically, a once-daily administration of 0.5% timolol corresponds to an approximate dose of 300 μg, representing less than 3% of the standard oral dosage (20 mg) (25). Nevertheless, the potential for systemic absorption raises valid concerns regarding fetal bradycardia and neonatal respiratory distress, which must be weighed against the benefits. While BBs are frequently utilized, a comprehensive safety assessment requires the examination of alternative agents. Both Hashimoto et al. and Pellegrino et al. reported a lack of association between PB (gestational age <37 weeks) and the use of PGAs and BBs (16, 20). Furthermore, the area under the concentration–time curve (AUC) for latanoprost free acid following topical administration is reported to be approximately 5–6 pg.·h/mL, a value substantially lower than the 400–700 pg.·h/mL range associated with abortion induction (26). The dosage required to induce abortion corresponds to approximately 400 mL of the ophthalmic formulation of latanoprost (27). This pharmacokinetic evidence suggests that the theoretical risk of miscarriage from topical therapy is negligible. However, the uterotonic properties of PGAs raise a theoretical concern that they may precipitate uterine contractions, potentially leading to early labor or preterm delivery (28).

Conversely, alpha-adrenergic agents pose documented risks of central nervous system and respiratory depression in infants and young children, a vulnerability attributed to the increased permeability of the blood–brain barrier in this pediatric population (29). Specifically, brimonidine has been shown to cross the placenta and may cause apnea or hypotonia in newborns, leading many experts to recommend its avoidance, especially in the third trimester. Given the potential risks associated with systemic absorption, strategies for IOP management should prioritize measures to mitigate this effect. Zimmerman et al. reported that the implementation of nasolacrimal occlusion or transient eyelid closure significantly mitigated systemic drug absorption, resulting in a reduction of up to 60% (30). Ultimately, clinical evidence suggests favorable safety profiles for key drug classes. Hashimoto et al. reported that CA was not associated with exposure to BBs, PGAs, or other glaucoma pharmacotherapies (16).

In clinical practice, glaucoma medications are prescribed to patients with more severe ocular hypertension or those at higher risk of glaucomatous progression (25). These patients may also have a higher incidence of maternal comorbidities (e.g., hypertension and diabetes) that are independently associated with adverse birth outcomes (17). Although we extracted and adjusted for available covariates where reported, the included observational studies did not consistently control for disease severity or comprehensive maternal risk profiles. Therefore, the observed association between glaucoma medication exposure and birth outcomes may be partially or wholly attributable to underlying maternal and ocular disease severity rather than a direct causal effect of the medications themselves. Future prospective studies with detailed characterization of baseline disease severity and maternal health status are needed to disentangle these effects.

This meta-analysis revealed that maternal exposure to antiglaucoma medications during pregnancy is significantly associated with an increased risk of LBW (OR = 2.12, 95% CI = 1.46 to 3.08). However, no statistically significant associations were observed between medication exposure and the risks of PB (OR = 1.58, 95% CI = 0.81 to 3.07) or CA (OR = 1.07, 95% CI = 0.24 to 4.77). These findings align with those of previous studies indicating that compared with women in the control group, women who were prescribed topical antiglaucoma medications had a significantly elevated risk of delivering LBW infants (17, 31). Following antiglaucoma medication exposure, the uterine vasculature tends to constrict, resulting in a reduction in uteroplacental blood flow. This placental insufficiency directly diminishes the delivery of oxygen and essential nutrients to the fetus, thereby restricting fetal growth (6). Given these findings, the decision to treat must be individualized, considering the trimester-specific risks and the severity of the glaucoma. Therefore, it is imperative for clinicians to discuss fertility intentions with women of reproductive age prior to conception and to provide comprehensive counseling regarding the risk–benefit profiles of various therapeutic modalities. A shared decision-making model, involving both the patient and a multidisciplinary team, is essential for navigating this complex landscape.

## 5 Limitation

This study is subject to several limitations that warrant consideration. First, the analysis relies heavily on observational data, as the majority of the included studies were retrospective cohort studies and case series. The inherent selection bias and confounding factors associated with these nonrandomized designs may influence the validity of the pooled estimates. The absence of randomized controlled trials limits the ability to establish a definitive causal relationship between antiglaucoma medication exposure and adverse birth outcomes. Second, the relatively small number of included studies limits the statistical power of our analysis. Under these conditions, we were unable to reliably assess publication bias using funnel plots or perform comprehensive meta-regression to explore the sources of heterogeneity. Third, all included studies were published between 2004 and 2024. While the inclusion of these studies offers valuable historical evidence regarding the safety of antiglaucoma medications, their generalizability to contemporary clinical practice must be interpreted with caution. Furthermore, studies evaluating multiple antiglaucoma medication classes yielded distinct exposure subgroups with shared control groups. While this prevents data omission, it causes a unit-of-analysis error through double-counting controls. Finally, we have formally assessed the certainty of the evidence using the GRADE methodology. The assessment indicates that the overall quality of evidence is low to very low. Despite these limitations, the robust findings regarding LBW provide valuable insights into the potential risks associated with antiglaucoma medications during pregnancy.

## 6 Conclusion

Our analysis suggests a potential association between maternal use of antiglaucoma medications and an increased risk of LBW. However, evidence regarding the risks of PB or CA remains inconclusive, as no statistically significant associations were observed. Additionally, maternal age was identified as a potential source of heterogeneity for CA. However, the preservation of maternal visual function remains a critical priority in certain cases. The goal of management during gestation is to mitigate glaucomatous progression through temporizing measures. Consequently, clinicians must strike a careful balance between ensuring maternal therapeutic efficacy and preserving fetal safety. Given the limitations of the included studies, these findings should be interpreted with caution, and further large-scale prospective studies are needed to confirm these associations.

## Data Availability

The original contributions presented in the study are included in the article/Supplementary material, further inquiries can be directed to the corresponding author.

## Supplementary material

SUPPLEMENTARY FIGURE S1 Risk of bias assessment based on the Joanna Briggs Institute (JBI) critical appraisal checklist for the 3 case series.

SUPPLEMENTARY FIGURE S2 Subgroup analyses for the risk of CA according to **(A)** mean maternal age, study design **(B)** and antiglaucoma agent **(C)**.
