# Supplementary figures and images for: Birth outcomes associated with maternal antiglaucoma medication exposure: a systematic review and meta-analysis
Source: Front Med (Lausanne). 2026 Jul 8;13:1872415. doi: 10.3389/fmed.2026.1872415 (PMC13388244; doi:10.3389/fmed.2026.1872415)

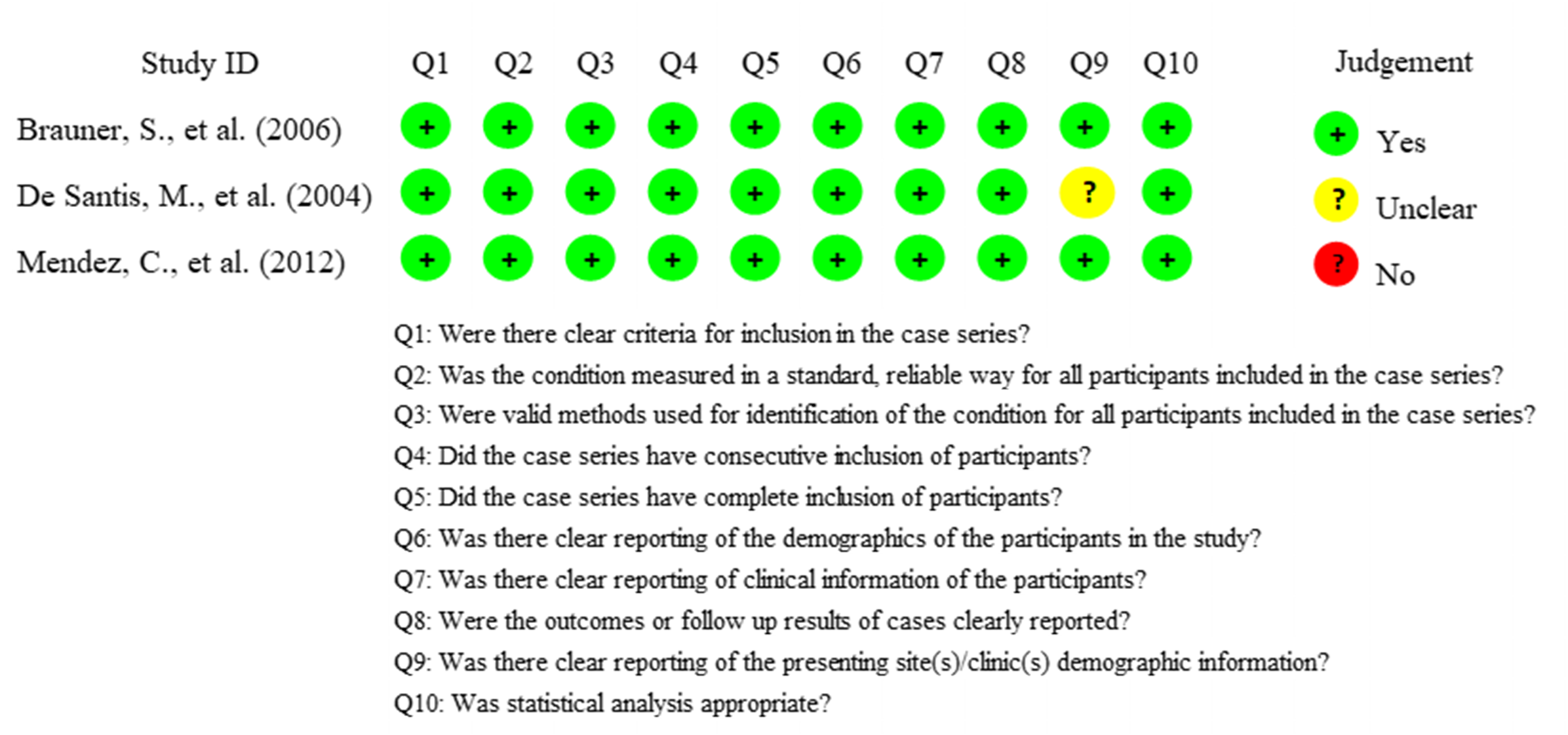

Supplement: SUPPLEMENTARY FIGURE S1 — Risk of bias assessment based on the Joanna Briggs Institute (JBI) critical appraisal checklist for the 3 case series. [file Image_1.tif]

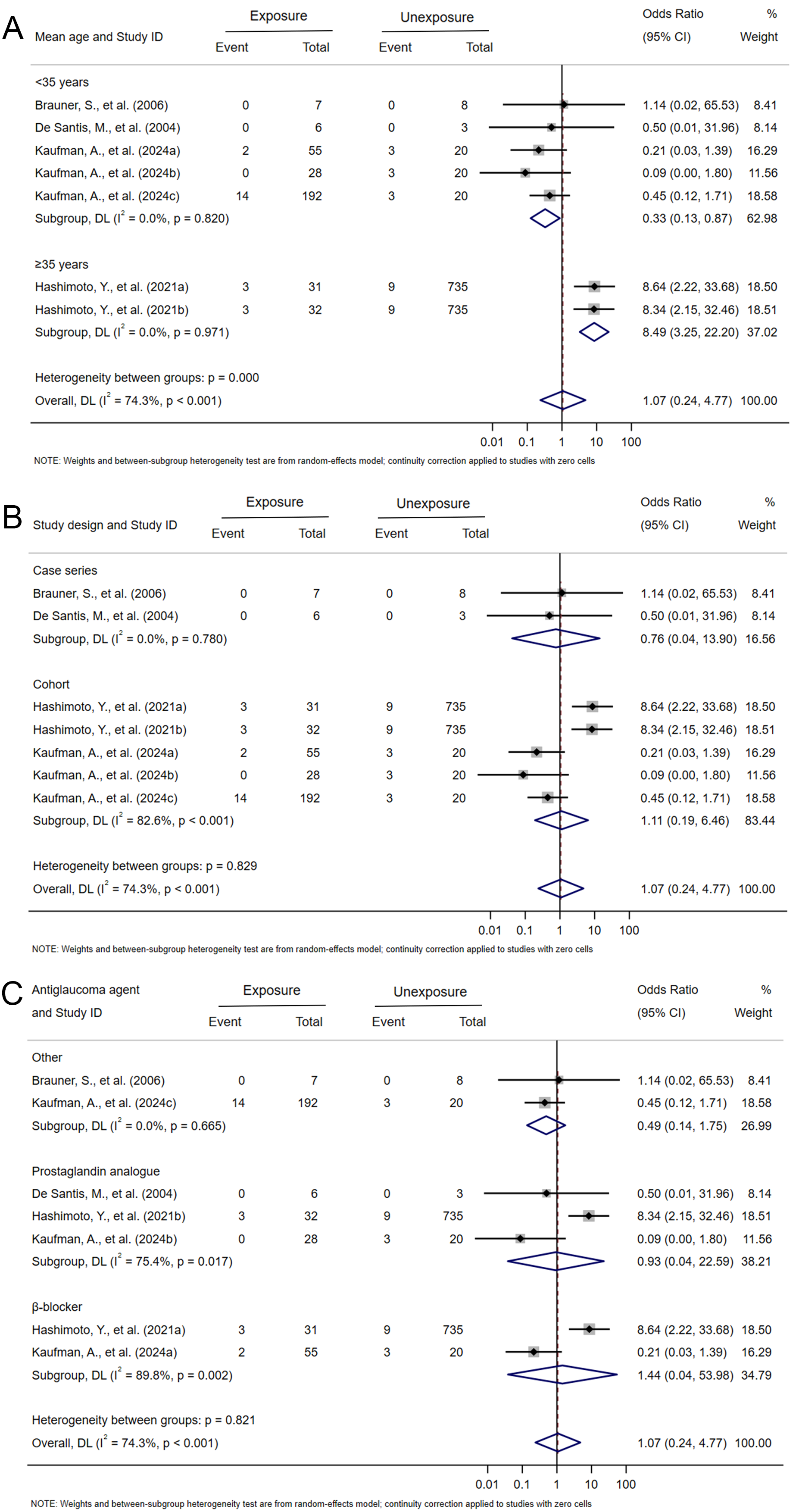

Supplement: SUPPLEMENTARY FIGURE S2 — Subgroup analyses for the risk of CA according to (A) mean maternal age, study design (B) and antiglaucoma agent (C). [file Image_2.tif]
