# Supplementary material for: Birth outcomes associated with maternal antiglaucoma medication exposure: a systematic review and meta-analysis
Source: Front Med (Lausanne). 2026 Jul 8;13:1872415. doi: 10.3389/fmed.2026.1872415 (PMC13388244; doi:10.3389/fmed.2026.1872415)
Supplement: Supplementary file 3 [file Table_1.docx]

Table S1. Search strategy for the databases.

| Databases | Search strategy | Published date | Results |
| --- | --- | --- | --- |
| PubMed | (Glaucoma OR Glaucomas) AND (Pregnancy OR Pregnant OR Gestation OR Gestational OR Pregnancies) | 1948-2026 | 525 |
| Web of science | Glaucoma OR Glaucomas (All Fields) AND Pregnancy OR Pregnant OR Gestation OR Gestational OR Pregnancies (All Fields) | 1900-2026 | 300 |
| ScienceDirect | ((Glaucoma or Glaucomas) and (Pregnancy or Pregnant or Gestation or Gestational or Pregnancies)) | 1997-2026 | 1527 |
| EMBASE | ((Glaucoma or Glaucomas) and (Pregnancy or Pregnant or Gestation or Gestational or Pregnancies)).af. | 1974-2026 | 1213 |
