# Supplementary material for: Birth outcomes associated with maternal antiglaucoma medication exposure: a systematic review and meta-analysis
Source: Front Med (Lausanne). 2026 Jul 8;13:1872415. doi: 10.3389/fmed.2026.1872415 (PMC13388244; doi:10.3389/fmed.2026.1872415)
Supplement: Supplementary file 4 [file Table_2.doc]

| Table S2. Quality assessment based on the Newcastle ‒ Ottawa Scale (NOS) checklist for the 5 cohort studies. | | | | | | | | | | |
| --- | --- | --- | --- | --- | --- | --- | --- | --- | --- | --- |
| **Study** | **Selection** | | | |  | **Comparability** |  | **Outcome** | | **Total score** |
|  | Representativeness of the sample | Sample | Non-respondent | Ascertainment of the exposure |  | Confounding factors are controlled |  | Assessment of outcomes | Statistical test |  |
| Hashimoto, Y., et al. (2021) | * | * | * | * |  | ** |  | * | * | 8 |
| Ho, J., et al. (2009) | * | * | * | * |  | * |  | * | * | 7 |
| Kaufman, A., et al. (2024) | * | * | * | * |  | ** |  | * | * | 8 |
| Pellegrino, M., et al. (2018) | * | * | * | * |  | * |  | * | * | 7 |
| Razeghinejad, M., et al. (2010) | * | * | * | * |  | * |  | * | * | 7 |
